# Transcription Factor SP2 Enhanced the Expression of *Cd14* in Colitis-Susceptible C3H/HeJBir

**DOI:** 10.1371/journal.pone.0155821

**Published:** 2016-05-18

**Authors:** Nils-Holger Zschemisch, Inga Brüsch, Anne-Sophie Hambusch, André Bleich

**Affiliations:** Institute for Laboratory Animal Science and Central Animal Facility, Hannover Medical School, Hannover, Germany; University of Saarland Medical School, GERMANY

## Abstract

Genetic analysis in the IL10-deficient mouse model revealed a modifier locus of experimental inflammatory bowel disease (IBD) on chromosome 18, with the allele of the strain C3H/HeJBir (C3Bir) conferring resistance and the allele of C57BL/6J (B6) conferring susceptibility. Differential *Cd14* expression was associated with this background specific susceptibility to intestinal inflammation. Polymorphisms of the *Cd14* promoter were found to be likely causative for strain specific expression, and *Cd14*-knockout mice revealed a protective role of this gene-product in experimental IBD. In this study, luciferase reporter assays confirmed an increased activity of the C3Bir derived *Cd14* promoter compared to the one of B6. Promoter truncation experiments and site-directed mutagenesis in both strains resulted in reduced *Cd14* promoter activity and confirmed that a central AP1 and the proximal SP1 transcription factor binding sites mediated the basal activity of the *Cd14* promoter in the mouse. Moreover, a T to C exchange at position -259 replaced putative STAT1 and CDX1 sites in the *Cd14* promoter from B6 by a SP2 site in C3Bir. Ablation of the Sp2 site through truncation was associated with a decreased promoter activity. Site-directed mutagenesis also demonstrated that the inactivation of SP2 led to a substantial loss of promoter activity in C3Bir. Performing electrophoretic mobility shift and supershift assays demonstrated interaction of SP2 with its potential binding site. In addition, retroviral—mediated overexpression of the SP2 transcription factor in primary bone marrow macrophages derived from C3Bir mice caused a significant increase in *Cd14* transcription. These data characterized SP2 as important factor responsible for higher *Cd14* expression and reduced IBD susceptibility mediated by the C3Bir allele.

## Introduction

*CD14* was identified as a marker and modifier gene in human IBD [1–3] and found to be associated to experimental IBD in the IL10-deficient mouse model [4–7]. CD14 is synthesized in a soluble (sCD14) and a membrane bound (mCD14) form. mCD14 is expressed on mature macrophages and bound to the cell membrane by a glycosylphosphatidyl-inositol (GPI) anchor [8]. Together with lymphocyte antigen 96 and toll like receptor 4 (TLR4) mCD14 forms a receptor for lipopolysaccharides (LPS); sCD14 circulates as a plasma protein due to a missing GPI anchor which is either not synthesized or it is post-translational detached by matrix metalloproteinase [9,10]. sCD14 competes with the membrane bound form for LPS and has an inhibiting effect on the activation of the complement system [11]. Less is known about the molecular mechanisms controlling expression and post-translational editing of soluble or membrane bound CD14. Landmann and co-workers demonstrated that Interferon-γ and IL4 down-regulate sCD14 expression in human monocytes and macrophages *in vitro* [12]. Synthesis of mCD14 and TLR4 is increased in patients with IBD and causes an enhanced reactivity to microbiota of the gut compared to healthy persons [13,14]. Regulatory mechanisms of *CD14* expression were studied in mouse, rat and man. Methylation is increased in the *CD14* promoter during childhood, inversely corresponding with reduced sCD14 levels [15]. Furthermore, promoter elements in the 5’UTR and flanking region determine molecular mechanisms controlling *CD14* expression [4,16–18|. Several AP1 binding site were identified in the *CD14* promoter in cow, mouse, rat and man mediating basal transcription activity [16–19]. SP1 regulatory elements found in the distal part of rat and human *CD14* promoter also contribute to basal promoter activity, but SP1 sites closely adjacent to the transcription start are responsible for reduced *CD14* transcription [17,18]. Furthermore, single nucleotide polymorphisms (SNP) modulated *CD14* activity in cow [19–21]. Human *CD14* promoter polymorphisms were involved in sensitization to allergens [22], atopic dermatitis [23], cardio-vascular diseases [24–27], tuberculosis and HIV infection [28–30], acute diarrhoea [31] and IBD [32–34]. Particularly the tantamount polymorphism C-260T/C-159T was found in patients with Crohn’s disease and ulcerative colitis [32,33,35–37].

We have identified *Cd14* as a major candidate gene for experimental IBD in mice by performing genome wide linkage analysis and subsequent microarray studies [5]. For these analyses interleukin-10 deficient (*Il10*^*tmCgn*^, *Il10*^*-/-*^) mice were used that developed intestinal inflammation spontaneously in a microbiome dependent manner. In this model system, expression of disease depended on the mouse background strain. Quantitative trait locus analysis revealed at least ten loci that contributed to IBD susceptibility in *Il10*^*-/-*^ mice on a C3Bir vs B6 background. A B6-derived susceptibility locus (*Cdcs6*) determined low intestinal expression of *CD14*, while the C3Bir allele of *Cd14* encoded high gene expression. *In silico* analysis of the *Cd14* promoter in B6 as well as C3Bir demonstrated differences in transcription factor binding site equipment and co-location. Polymorphisms introduced STAT1 and SP2 binding sites in the *Cd14* promoter of C3Bir mice while in B6 animals BCL6 and PPARγ elements were additionally present [4]. Therefore, the aim of this study was to identify regulatory factors that are associated with *Cd14* promoter polymorphisms and thereby causing both strain specific *Cd14* expression and disease susceptibility. Protective factors may serve as targets for prospective novel therapeutic approaches for IBD.

## Materials and Methods

### Identification of transcription factor binding sites

The *Cd14* promoter sequence from -1067 to +199 in B6 and -1067 to +203 in C3Bir as well as the human *Cd14* promoter (GenBank: X74984 and U00699) were analyzed to identify putative transcription factor binding sites using the MatInspector Software (Genomatix Software GmbH, Munich, Germany).

### Cell culture

RAW264.7 cells (ATCC: TIB-71, murine macrophages) were grown in Dulbecco’s Modified Eagle Medium (DMEM, Life Technologies, Darmstadt, Germany), supplemented with 10% FCS (PAA, Pasching, Austria) and 5% Penicillin/Streptomycin (Biochrom, Berlin, Germany). When cells reached a confluence of 70% they were scraped and split 1:4. NIH3T3 cells (ATCC: CRL-1658, murine embryonic fibroblasts) were grown in DMEM with GlutaMax^™^ (Life Technologies), supplemented with 10% FCS (PAA) and 5% Penicillin/Streptomycin (Biochrom). Every third day or at reaching a confluence of 50% cells were split 1:10. L929 cells (murine fibroblasts derived from C3H/An mice) were seeded at 5 x 10^5^ on 75 cm^2^ cell culture flasks and cultured in 55 ml DMEM (high glucose, GlutaMAX^™^ Supplement, pyruvate) supplemented with 10% FCS and 5% Penicillin/Streptomycin for 10 days [38]. Medium was harvested, filtered through a 45μm filter system and transferred to 50 ml tubes. L929 conditioned medium was stored at -20°C.

### Isolation of primary bone marrow macrophages (BMM)

Bone marrow of tibia and femur from 6 C3Bir mice were flashed out using DMEM. The bone marrow suspension were filtered through a 70 μm cell strainer and cells were centrifuged at 1000 rpm for 10 minutes at 4°C. The bone marrow cells were resuspended in BMM medium (DMEM, high glucose, GlutaMAX^™^ Supplement, pyruvate supplemented with 10% FCS, 10% L929- conditioned medium, 5% Penicillin/Streptomycin) and seeded on a dozen 6 well plates. After four hours of culture BMM became adherent and medium was changed [38]. All cells were incubated with 5% CO_2_ at 37°C.

### Production of Moloney Murine Leukemia Virus (MMULV)-based retroviruses

6 x 10^6^ amphotrophic Phoenix-Ampho retrovirus producer cells were seeded on 10 cm cell culture dishes in DMEM (high glucose, GlutaMAX^™^ Supplement, pyruvate, Gibco 10569010) and grown overnight to a confluence of 80–90%. 32μg of the plasmid pBABE puro-Sp2 encoding for the murine SP2 transcription factor [39] or of the empty backbone vector pBABE puro were transiently transfected using 50 μl of Lipofectamine 2000 (Invitrogen) following the manufacturer’s instructions. 24 and 48 hours after transfection the cell culture supernatant were harvested, centrifuged at 1200 rpm for 10 min at 4°C and the retroviral suspension was stored at -80°C.

### Retroviral transduction

After 4 days of BMM culture the medium was changed to 3 ml fresh BMM medium. Cells were transduced with 200 μl retroviral suspension and 6μg/ml Polybrene (Merck Millipore, Billerica, MA) and centrifuged for 1 hour at 200xg at room temperature. After transduction infected BMMs and untreated control cells were cultured for additional 48 hours.

### Quantitative Real-time gene expression analysis

After RNA isolation from BMM with the RNeasy Plus Mini Kit (Qiagen, Hilden, Germany) 500 ng of total RNA were reverse transcribed with the QuantiTect Reverse Transcription Kit (Qiagen) following the supplier’s recommendations. 1 μl of the cDNA, the TaqMan Fast Advanced 2x Master Mix (Applied Biosystems, Fisher City, CA, USA) and a *Cd14* TaqMan Gene Expression Assay (Mm00624086_cn, Applied Biosystems) were used to determine *Cd14* expression with the StepOnePlus Real-Time PCR System (Applied Biosystems, Darmstadt, Germany). The expression of *Actb* as endogenous control was measured using a TaqMan Gene Expression Assay (Mm00607939_s1, Applied Biosystems). Results were statistically analysed performing unpaired t-test with the Prizm 5 software (GraphPad Software, LaJolla, CA). Significance levels were set to p < 0.05.

### Protein extraction and western blotting

The two cell lines were tested for their basal *Cd14* expression and expression after stimulation with LPS (0.1 ng/ml, Sigma-Aldrich, Munich, Germany) for 12 hours. Proteins were isolated from the cells by using NP40 buffer (1 mM PMSF, 1 mM DDT, 1 mM sodium ortho-vanadate, Complete Mini Protease inhibitor cocktail tablet [Roche Applied Science, Mannheim, Germany]) and concentrations were defined by performing Bradford assays. 50 μg protein were used for SDS-PAGE followed by a semi-dry transfer to Optitran BA-S 85 membrane (Sigma-Aldrich, Munich, Germany) using Semi-Dry Blotter Maxi V20-SDB (Carl Roth, Karlsruhe, Germany) and transfer buffer (39 mM glycine, 0.037% SDS, 48 mM Tris, 20% methanol). Primary antibodies were applied to bind to CD14 (rat monoclonal [Sa14-2] to CD14 [FITC], Abcam, Cambridge, UK) or to beta actin (mouse monoclonal [mAbcam 8226] to beta Actin, Abcam). After labeling CD14 antibody with goat anti rat IgG [HRP] (AP136P; Millipore, Billerica, USA) and beta actin with F(ab’) 2 rabbit anti mouse IgG [HRP] (STAR21B; AbD serotec, Oxford, UK) as secondary antibodies, luminescence of the different bands was activated by using Roti-Lumin 1 and 2 solution (Carl Roth). Detection of the bands was performed by x-ray film (GE Healthcare, Munich, Germany)

### *Cd14* promoter truncations

Promoter truncations were prepared by PCR using Phusion Hot Start Polymerase and 5xHF-Buffer as preset by the manufacturer (NEB, Ipswich, UK), utilizing primers and annealing temperatures depicted in Supporting Information (S1 Table). Linearized pGL4.17 plasmid (Promega, Wisconsin, USA) containing *Cd14* full-length promoters of C3Bir respectively B6 were served as templates [4]. For quality control the promoter fragments of C3Bir were flanked by *KpnI* and *BglII*,while B6 elements were flanked by *XhoI* and *BglII* restriction sites. Cloning of the truncated promoter elements was performed using StrataClone PCR Cloning Kit (Agilent, San Diego, CA) according to the suppliers recommendations. The promoter sequences were subsequently excised from pSC-A amp/kan with *XhoI*/*BglII* or *KpnI*/*BglII* (NEB) and inserted into *XhoI*/*BglII* or *KpnI*/*BglII* digested luciferase reporter plasmid pGL4.17.

### Site-directed mutagenesis

Mutagenesis of both full-length promoters in pGL4.17 was performed using Phusion Site-Directed Mutagenesis Kit (Biozym, Hessisch Oldendorf, Germany) according to the manufacturers recommendations with minor modifications. Therefore short base pair exchanges or insertion of *Asc*I or *Pme*I restriction endonuclease recognition site were introduced into the core sequence of the putative restriction factor binding sites. Primer sequences are depicted in Supporting Information (S2 and S3 Tables). After digestion with the methylation sensitive *DpnI* (NEB) to eliminate methylated templates, PCR products were ligated and transformed into XL10-Gold Kan multicompetent cells (Agilent).

### Sequencing

Amplification products were sequenced using the Big Dye Terminator v1.1 Cycle Sequencing Kit (Invitrogen, Darmstadt, Germany) with T7, T3 or *Cd14* specific primers for 25 cycles (96°C for 30 s, 60°C for 4 min). Reaction clean up was performed using innu-PREP-DYEpure-Kit (Analytic Jena, Jena, Germany) and sequencing was run at ABI PRISM 310 Genetic Analyzer (Applied Biosystems, Carlsbad, CA). Alternatively samples were sent to MWG Eurofins Operon (Ebersberg, Germany).

### Transfection efficiency test

XtremeGene HP DNA, XtremeGene 9 DNA (Roche Applied Science, Mannheim, Germany), Superfect and Polyfect Transfection Reagents (Qiagen, Venlo, Netherlands) were tested to achieve the highest efficiency by co-transfection of 2,5 μg pAdTrack-CMV and 2,5 μg plasmids CMV-β-Gal following the manufacturer’s instructions into RAW264.7 cells. 24 h and 48 h after transient transfection RAW264.7 cells were harvested using extraction buffer to determine transfection efficiency with a β-galactosidase assay [4].

### *Cd14* promoter activity

RAW264.7 cells were co-transfected with 4 μg promoter constructs containing pGL4.17 luciferase reporter plasmid and 1 μg CMV-β-Gal plasmid using Polyfect Transfection Reagent. Promoter activity and transfections efficiency were evaluated by performing luciferase and β-galactosidase assays on harvested cell extracts as described earlier [4]; luminescence was measured using 2030 Multilabel Reader Victor X3 (Perkin Elmer, Waltham, MA). Results were statistically analysed performing One-way Anova with Bonferroni’s Multiple Comparison Test with the Prizm 5 software (GraphPad Software, LaJolla, CA). Significance levels were set to p < 0.05.

### Electrophoretic mobility shift assay (EMSA)

Physical interaction between a transcription factor and their putative binding site were confirmed performing EMSA and Supershift Assay using the Gelshift Chemiluminescent EMSA Kit (Active Motif, Rixensart, Belgium). Nuclear extracts (NE) were isolated from RAW264.7 cells using the Nuclear Extract Kit (Active Motif) following the suppliers’ recommendations. 50 μg of either the biotinylated Sp2 forward (5’-GTTTTTCGTCCCTCCCTAAAAAACACT-3’) and reverse (5’-AGTGTT TTTTAGGGAGGGACGAAATTG-3’) consensus oligonucleotides were dissolved in 10 x annealing buffer (200 mM Tris, pH 7.6; 100 mM MgCl_2_; 500 mM NaCl) with Tris-EDTA buffer, ph 7.6 and heated for 5 min at 70°C. The double stranded oligonucleotide then cooled down to room temperature over night inside the heating block together with the unlabeled Sp2 oligonucleotides. For the binding reaction of the transcription factor to the consensus oligonucleotides 10 μg NE was incubated with 20 fmol of the biotinylated consensus oligonucleotide and for competitive binding with additional 2–8 pmol unlabeled consensus oligonucleotide in the reaction buffer (1x binding buffer, 2.5% glycerol, 50 ng/μl Poly d(I-C), 0.05% NP-40)for 20 min at room temperature. The Supershift Assay was performed by a pre-incubation of the NE with 2 μg of antibodies (Santa Cruz Biotechnology) either against SP1 (H-225; sc-14027 X), SP2 (K-20; sc-643 X) or SP3 (D-20; sc-644 X) for 30 min on ice. Protein DNA complexes were separated electrophoretically in a 6% bisacrylamide TBE mini gel for 1 hour in 0.5% TBE buffer at 100 V followed by a semy-dry transfer (Semi-dry Blotter Model L; Phase, Lübeck, Germany) with 0.5% TBE buffer at 10 V for 15 min to the Hybond-N^+^ membrane (GE Healthcare, Little Chalfat, UK). After UV-crosslinking of the membrane at 312 nm for 15 min the detection of the mobility shifts were performed following the suppliers’ recommendations.

## Results

### Optimization of *Cd14* promoter analysis system

**Fig 1:**
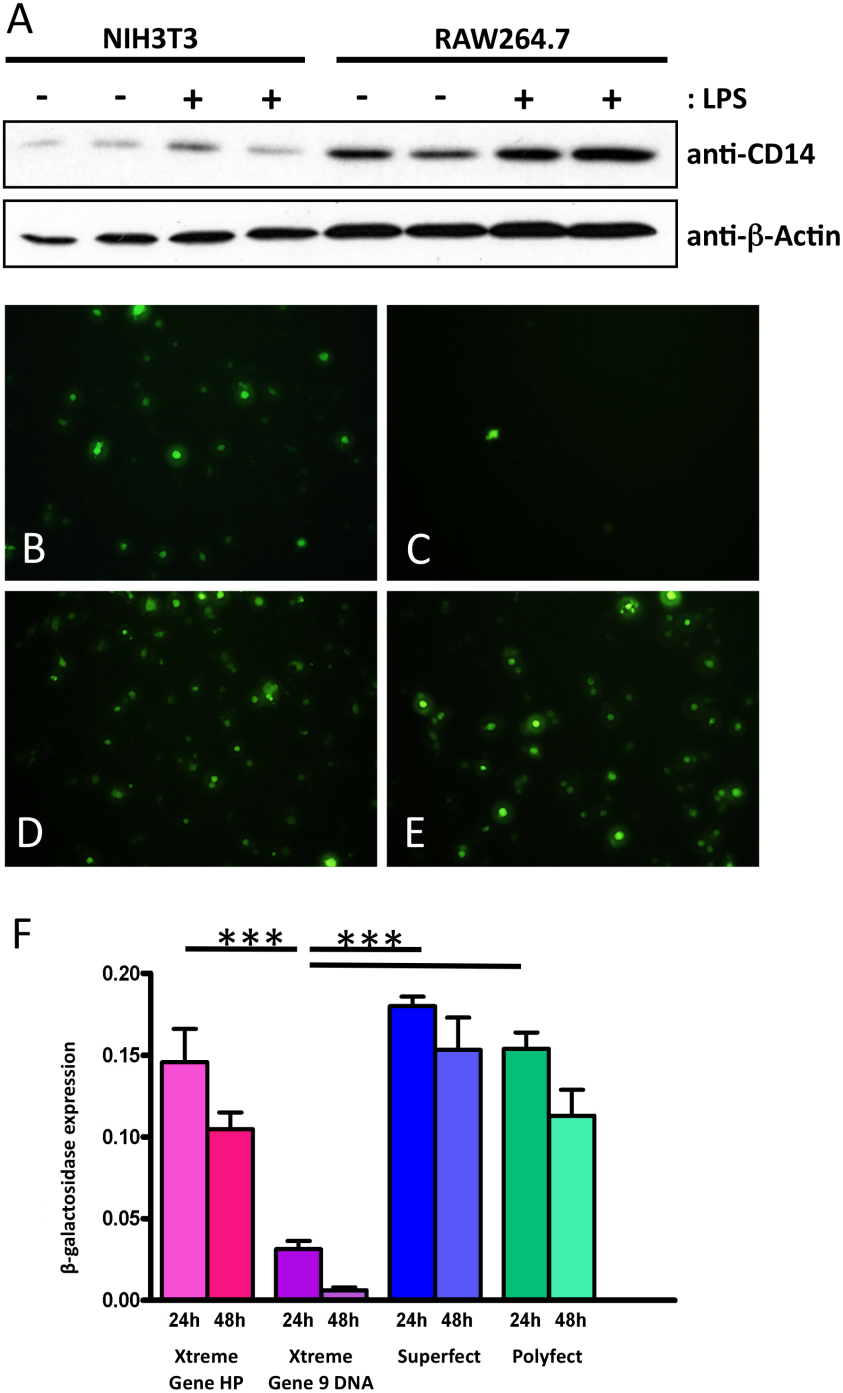
Transfection system establishment. **A** Western blot analysis of CD14 expression in untreated and LPS stimulated murine RAW264.7 macrophages and NIH/3T3 fibroblasts, β-actin as endogeous control. Transfection efficiency test by determination of GFP-positive RAW264.7 cells by cyto fluorescence 24 hours after transfection with **B** Xtreme Gene HP, **C** Xtreme Gene 9 DNA, **D** Superfect, **E** Polyfect, and **F** by β-galactosidase assay, ***: p < 0,05.

To establish a reproducible and efficient transfection system, a suitable cell line as well as an appropriate transfection reagent had to be identified. Western blot analysis revealed a considerably higher basal CD14 expression in RAW264.7 than in NIH3T3 cells (Fig 1A). LPS stimulation did not show any effect on CD14 expression in NIH3T3 in contrast to the increased CD14 protein concentration in RAW264.7 cells. In addition, various chemical transfection methods were tested on RAW264.7 cells using the plasmids pAd-Track-CMV and pCMV-βGal to ensure reproducible high transient transfection efficiencies. GFP fluorescence and β-galactosidase assay revealed a significant lower expression level using XtremeGene 9 DNA (Fig 1C and 1F) after 24h and 48h after transfection compared to Superfect, Polyfect or XtremeGene HP transfection reagents achieving an high equal expression level (Fig 1B, 1D, 1E and 1F). Therefore, Superfect transfection reagent was chosen as tool for transient transfection of reporter plasmids into RAW264.7 cells.

### SNPs changed the patterns of putative transcription factor binding sites

**Fig 2:**
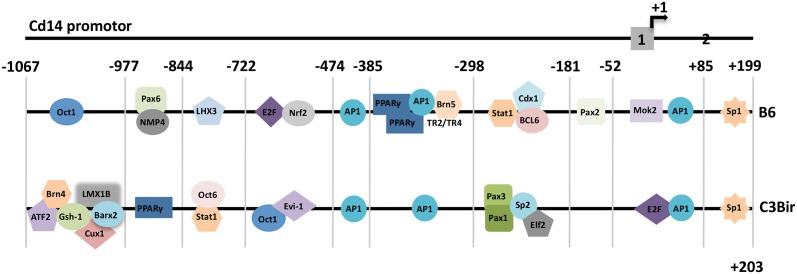
Patterns of transcription factor binding sites associated with inflammation and colitis. Bioinformational analysis of the *Cd14* alleles from the B6 and the C3Bir strain revealed striking changes in transcription factor binding sites caused by SNPs. AP1 and Sp1 sites were common in both alleles contributing to the control of *Cd14* expression in mice; +1: translation start site; grey boxes: exons 1 and 2.

In promoter sequences of C3Bir and B6, identical recognitions sites for AP1 (-417 to -407, -367 to -356, + 3 to +14) and SP1 (+141 to +156) were identified by bioinformational analysis of putative transcription factor binding sites (Fig 2). However, polymorphisms in the *Cd14* promoter sequences induced strain specific recognition sequences in C3Bir and B6 (Fig 2). We identified a cluster of alternative sites for ATF2 (-1055 to -1038), BRN4 (-1044 to -1026), GSH-1 (-1041 to -1025), CUX1 (-1040 to -1022) LMX1B (-1040 to -1026) and BARX2 (-1039 to -1022) caused by the exchanges from T to C at position -1041 and from G to A at position -1034 in the C3Bir allele, while in the B6 allele a binding site for OCT1 was found covering the sequence -1044 to -1030 (Fig 2). The C to T substitution at position -876 introduced a PPARγ site (-878 to -856) in the C3Bir allele instead of binding motifs for NMP4 (-885 to -875) and PAX6 (-885 to -867) found in the *Cd14* promoter of B6. Additionally, a LHX3 site (-824 to -810) in the B6 *Cd14* promoter was replaced in the C3Bir allele by an OCT6 (-835 to -823) site and STAT1 sites (-833 to -815; -832 to -830) caused by an A to G exchange. The loss of E2F (-601 to -585) and NRF2 (-601-581) found in the B6 allele caused by a C-596T polymorphism at position was compensated by the introduction of OCT1 (-606 to -592) and EVI-1 (-604 to -588) sites in the C3Bir allele of *Cd14*. The C-360T polymorphism introduced binding sites for PPARγ homodimers (-376 to -854), PPARγ-RXR heterodimers (-366 to -344), and NRC2c family members (-366 to -345) while the G-328T polymorphism generated an additional BRN5 site (-329 to -311) in the B6 allele. A cluster of alternative transcription factor binding motifs was found in the C3Bir allele containing PAX3 (-268 to -250), PAX1 (-267 to -249), SP2 (-244 to -229), and ELF2 (-255 to -235) caused by T to C and G to C substitutions at positions -260 and -244, respectively. However, in the complementary region of the *Cd14* promoter from B6 unique binding site for STAT1 (-266 to -248), CDX1 (-264 to -246), and BCL6 (-263 to -247) (Fig 2) were identified. Upstream of the modifications the C-75T polymorphism introduced a PAX2 binding motif (-75 to -53) in the B6 allele, while the C-49T exchange led to a MOK2 binding site (-50 to -30) in B6 eliminating the E2F site (-51 to -35) found in the C3Bir allele (Fig 2) [4].

### Truncations of the *Cd14* promoter

To figure out which binding sites were associated with the strain specific *Cd14* expression, the full-length promoter constructs of both strains were truncated to map regions essential for transcription regulation.

**Fig 3:**
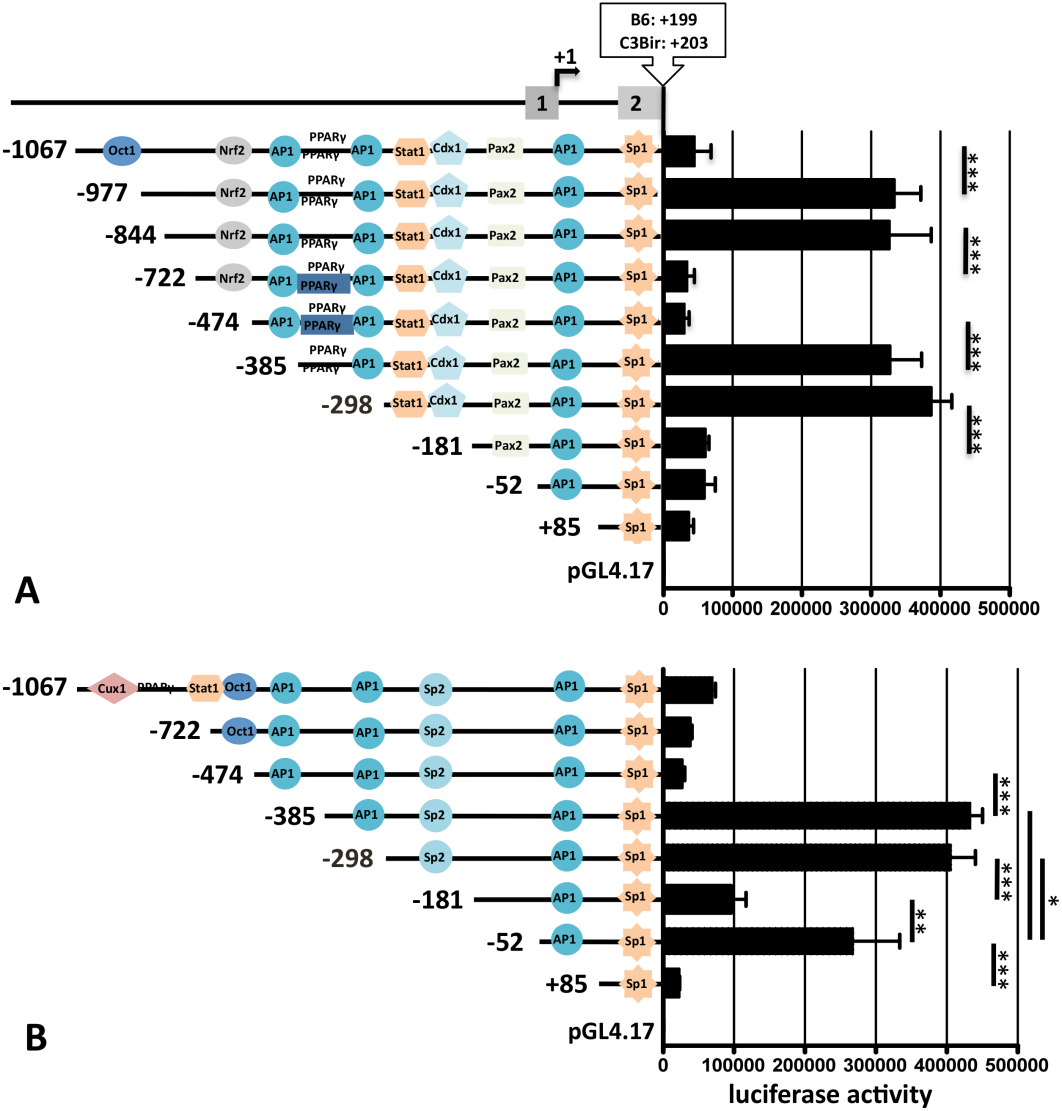
Truncations of the *Cd14* promoter alleles. Constructs of the 5’ truncated *Cd14* promoter were cloned in the pGL4.17 reporter plasmid and transfected in RAW264.7 cells. **A** The strong luciferase activity increase after removal of the distal 100 bp and of the sequence between -474 and -385 in the B6 allele indicated inhibitory elements in these regions. Activating binding motifs were identified through the drop of luciferase activity after cutting of the -844 to -722 and -298 to -181 regions. **B** Stepwise truncation of the C3Bir allele revealed significant expression peaks after removal of the -474 to -385 region and the -181 to -52 sequence while the -298 to -181 and the -52 to +85 regions seemed to contain stimulating binding motifs. ***: P < 0,05.

Luciferase reporter assay showed that 5’ truncation of the B6–1067/+199 full-length promoter of *Cd14* to -722/+199 increased promoter activity significantly sixfold to more than 3 x 10^5^ associated with the loss of OCT1 binding sites. Therefore, the distal part of the promoter contained a stimulating element. Further truncation to -844/+199 did not change the reporter expression (Fig 3A). Cutting off the region up to -474/+199 in the next steps reduced the luciferase transcription to the level of the full-length promoter. These results suggested that the sequence between -977 and -474 containing an NRF2 binding site has a stimulatory impact (Fig 3A). Continuing truncation to -385/+199 led to a reconstitution of the high reporter expression as detected in the -977/+199 and -844/+199 promoter constructs. This result was associated with the loss of the distal AP1 binding site while the removal of the PPARγ and proximal AP1 sites by reduction of the promoter to -298/+199 obtained no significant change in the reporter activity. After ablation of the -298 to -181 part of the promoter containing STAT1 and CDX1 the luciferase expression dropped again to 60177 counts (SD ± 11309) demonstrating the loss of an activating transcription factor binding site between -298 and -181. Further truncation to -52/+199 and +85/+199 induced only weak expression decreases compared to the -181/+199 promoter fragment (Fig 3A).

-1067/+203 full-length *Cd14* promoter showed a higher activity in the luciferase assay as reported previously [4]. In C3Bir the truncations to -722/+203 bp deprived of CUX1, PPARγ and STAT1 binding sites and to -474/+203 depleting an OCT1 binding motif resulted in a stepwise but not significant reduction of reporter activity (Fig 3B). Like in the B6 *Cd14* promoter allele the loss of the distal AP1 binding site by reduction to -385/+203 led to a strong increase of the reporter expression. The ablation of the proximal AP1 by cutting off the region between -385 and -298 did not change the luciferase activity significantly as seen in the B6 allele (Fig 3B). Further truncation to -181/+203 associated with the loss of the SP2 recognition motif led to strong decrease in reporter activity compared the the -385/+203 and -298/+203 constructs. Reduction of the C3Bir allele of the Cd14 promoter to -52/+203 resulted again in a strong increase of the luciferase activity suggesting an activation element between -181 and -52 while the deletion of the AP1 site at the 3’end shutted down the luciferase expression (Fig 3B).

These results implicated inhibiting functions of the distal OCT1 binding site in *Cd14* promoter of B6 and strong activating properties of the STAT1/CDX1 binding motif located between -298 and -181. The depletion of the region between -474 and -385 containing the distal AP1 site led in both promoter alleles to a significant increase in reporter activity. The drop of reporter activity induced by cutting off the region containing the SP2 binding site of the *Cd14* promoter of C3Bir demonstrated an stimulating influence of the -298 to -181 sequence. These experiment also confirmed that the proximal SP1 site regulated the basal activity of the both alleles [17, 18].

### Modulation of *Cd14* promoter activity through site-directed mutagenesis

**Fig 4:**
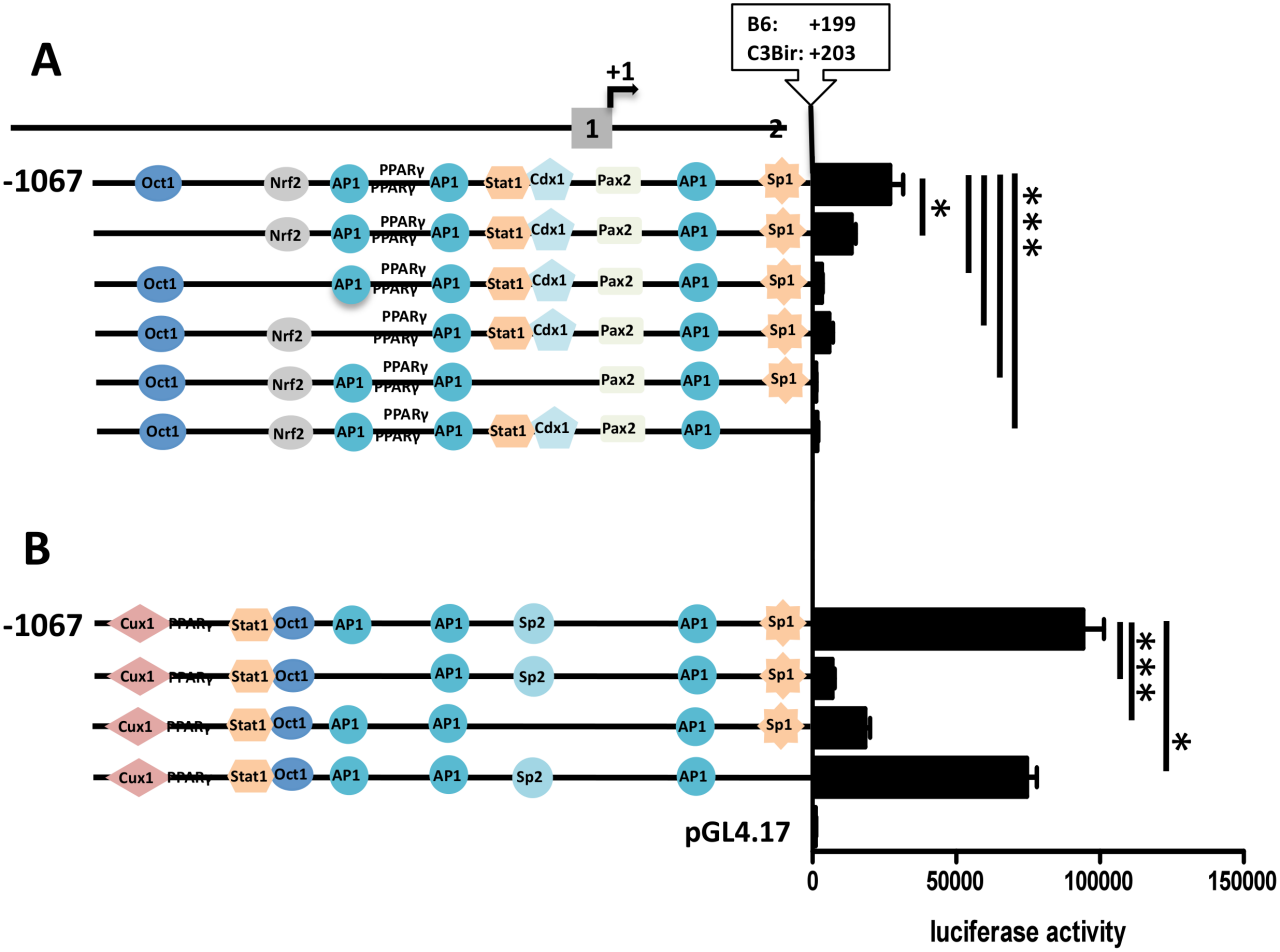
*Cd14* promoter activity after site-directed mutagenesis. **A** After inactivation of OCT1, Nrf2 and proximal AP1 binding sites in the B6 allele reduced activities were detected while in promoter constructs lacking Stat1/Cdx2 and SP1 led to complete transcriptional silencing. **B** In the C3Bir allele deletion of AP1 and SP2 binding sites resulted in significant transcriptional decrease, while after the mutation of SP1 only a slight reduction of Luciferase expression was seen. ***: P < 0,05.

To proof the predicted influence of the transcription factor binding sites on *Cd14* expression site-directed mutagenesis followed by luciferase reporter assays were performed. Mutagenesis of the distal Oct1 binding site in the full length promoter of the B6 allele induced a significant reduction of the reporter activity while destruction of the NRF2 binding motif led to a almost complete shut down of the Cd14 activity (Fig 4A). As expected, disruption of the distal AP1 binding site caused a strong decrease of reporter acitivity, while the simultaneous mutagenesis of the Stat1/CDX1 binding motifs and the ablation of the proximal SP1 site resulted in silencing of the B6 allele of the *Cd14* promoter (Fig 4A). As in the B6 allele, the mutagenesis of the distal AP1 site induced a significant drop of the promoter activity confirming the activating influence of AP1 (Fig 4B). The disruption of the SP2 binding site diminished reporter activity significantly to 10049 (SD± 2584) compared to the activity of wild type C3Bir allele with 89503 (SD± 13839). Like in the B6 allele of the *Cd14* promoter the experimental mutation of the 3’ SP1 site caused a reduction of the reporter activity (Fig 4B). These data suggested that the distal AP1 site and the proximal SP1 were responsible for the basal activity of the *Cd14* promoter in both mouse strains. The results demonstrated that the OCT1 binding site in the -1067 to -977 region of the B6 allele did not mediate the proposed inhibitory effect while the NRF2 binding motif had an unexpected activating effect. The predicted stimulating effect caused by the STAT1/CDX1 binding site in the B6 allele was confirmed by the mutagenesis experiment. However, the disruption of the SP2 recognition motif in the C3Bir allele induced to the expected reduction of promoter activity and led to the hypothesis that SP2 as stimulating factor was in charge for the higher expression level of *Cd14* in C3Bir compared to B6.

### Physical interaction of SP2 with the C3Bir allele of the *Cd14* promoter

**Fig 5:**
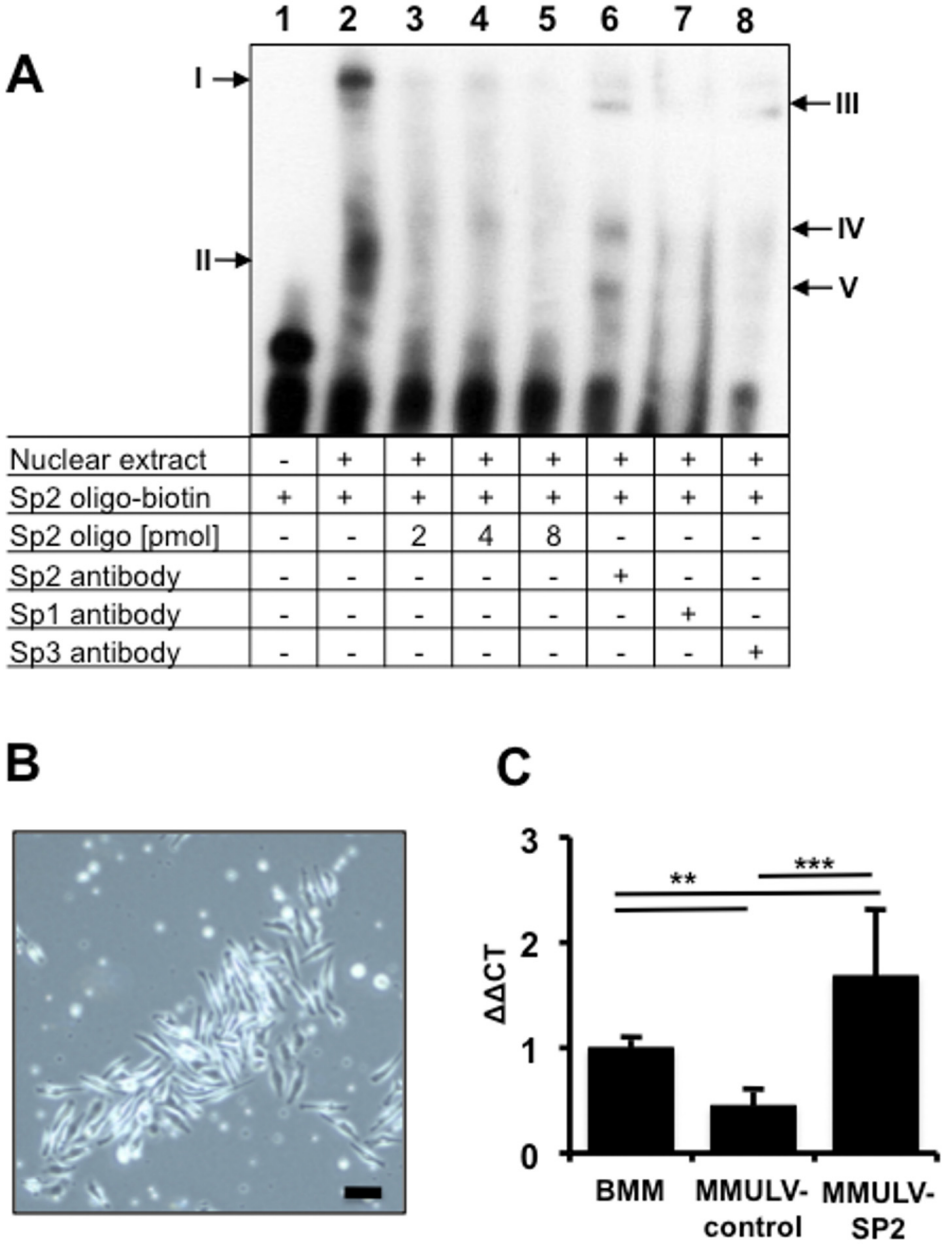
Physical interaction of SP2 with the Cd14 promoter derived from C3Bir. **A** SP2 electrophoretic mobility shift was performed running a biotin-labelled Sp2 consensus oligonucleotide alone (lane 1) or incubated with a nuclear extracts isolated from RAW264.7 cells (lane 2). In the latter two complex formations (I and II) from the Sp2 consensus oligo with nuclear extract components were detected. Competition of increasing amounts of unlabelled SP2 oligo for binding to the nuclear extract proteins led to a stepwise disappearance of biotin-Sp2-oligo protein complexes (lane 3–5). After pre-incubation of the nuclear extracts with an SP2 specific antibody new complexes (III-V) were established in this supershift assay demonstrating physical interaction of the SP2 protein with its consensus oligo (lane 6). Specific SP2 DNA complex formation was also demonstrated by the failure of SP1 and SP3 antibodies to bind to the protein DNA complexes (lane 7 and 8). **B** BMMs after four days of culture. The scale bar represents 10μm at a 10x magnification. **C** The MMULV-mediated overexpression of mouse SP2 in primary BMMs derived from C3Bir mice correlated with a higher Cd14 transcription rate compared to untreated and control virus infected macrophages. ***: p<0.01; ** p< 0.05

EMSA using a consensus oligonucleotide representing the transcription factor binding sites for SP2 derived from the C3Bir allele formed two complexes with the protein extracts isolated from RAW264.7 cells. In lane 2 of the EMSA (Fig 5A) the complex I remained in the upper part of the gel demonstrating the formation of a large protein-DNA aggregation. Also the faster migrating complex II was identified. Moreover, competitive application of unlabelled oligonucleotide abolished dose-dependent changes in migration shifts (Fig 5A, lanes 3–5). Incubation of oligonucleotide-protein complexes with an SP2 specific antibody induced formation of specific complexes III-IV (Fig 5, lane 6) witch were absent using SP1 or SP3 specific antibodies (Fig 5, lanes 7 and 8). Moreover, while the infection of proliferating primary BMMs *in vitro* (Fig 5B) with an MMULV-based control retrovirus reduced the expression of *Cd14* compared to untreated BMMs (Fig 5C) the retroviral-mediated overexpression of the mouse SP2 protein in primary BMMs caused a significant increase in *Cd14* expression in two independent experiments (Fig 5C). These results clearly revealed physical interaction between SP2 with the murine *Cd14* promoter.

## Discussion

The *Cd14* promoter of the mouse strains C3H/HeJBir and C57BL6/J display common transcription factor binding sites. Three AP1 and a single SP1 recognition site in close proximity to the transcription start site were identified. These results confirmed the work of Matsuura and colleagues who localized an AP1 binding elements in the *Cd14* promoter derived from BALB/c mice identical to AP1 site at -367 to -356 in the B6 and C3Bir alleles [16,19]. AP1 binding sites were also found in rat, cattle and human *CD14* promoter controlling the basic promoter activity like in the mouse [17,18]. Similarly, SP1 sites are well known regulatory elements in the *Cd14* promoter of different species [17–19]. The SP1 site in the mouse *Cd14* promoter in spatial proximity to the translation start site mediates basal promoter activity in both alleles revealed a reduced transcription rate after truncation to the shortest +85 fragment. In the human *CD14* promoter the truncation to the -128 fragment containing the proximal SP1 site also resulted in a sparse reduction of reporter activity [17], while the shortening of the rat *Cd14* promoter to the -142 construct including the 3’ end SP1 binding motif caused a drop of reporter expression to 20% compared to the full length promoter [18]. Moreover, the activating influence of the proximal SP1 site in the rat was confirmed by the significant lower reporter expression found after mutagenesis of the SP1 site in the full length promoter [18]. These data demonstrate that the 3’ SP1 site in rodents are associated with the maintenance of basal *Cd14* promoter activity in contrast to the inhibiting function in the human *CD14* promoter. Besides these corresponding features of the B6 and C3Bir allele we try to identify inhibiting transcription factor binding sites in the B6 allele that are lost or replaced by a stimulating binding motif in the C3Bir allele explaining the higher transcriptional level of *Cd14* in the C3Bir strain.

Truncation experiment revealed an inhibitory element in the distal -1067 to -977 region of the B6 allele containing an OCT1 binding site that was replaced by a CUX1 binding motif in the C3Bir allele through a T to C exchange at position -1041. Mutagenesis of this OCT1 consensus sequence demonstrated clearly that the OCT1 site has activating functions. The stimulating effects of OCT1 were already shown on B cells and in the expression of *Lipoxin A4* in human monocytes [40,41]. Therefore, the activation by OCT1 must be superimposed by yet unkown repressive elements in the -1067 to -977 region. Also the NRF2 binding site between -722 and -474 of the B6 allele of *Cd14* mediates transcriptional activation revealed by the transcriptional down-regulation after mutagenesis. On the other hand the elemination of the sequence containing the NRF2 site by truncation did not changed reporter activity suggesting that also in the -722 to -474 region of the B6 allele of the cd14 a unkown inhibitory element seems to neutralize NRF2 stimulation. Overexpression of NRF2 (Nuclear factor (erythroid-derived 2)-like 2) in rat alveolar macrophages after transient tranfection in vitro increased the Cd14 protein level while the expression of an anti-NRF2 siRNA accomplished the opposite effect [42]. NRF2 supported the maintenance of the intestinal barrier and reduced the colonic inflammation in chronic kidney disease [43]. Moreover, NRF2-deficient mice have been shown to be more susceptible to dextran sulfate sodium (DSS) -induced colitis than wild type animals [44] suggesting that expression of the protective the Cd14 protein and therewith the colitis manifestation in B6 mice was modulated by the NRF2 transcription factor.

Following elimination of the *Cd14* promoter region from the B6 allele containing the STAT1/CDX1 transcription factor binding site between -298 and -181 as well as after the mutagenesis of these binding motifs the reporter expression was diminished emphasizing the stimulatory function of STAT1 or CDX1. The transcription CDX1 was characterized as an intestine-specific homeoprotein but in contrast to the hypersensitivity to DSS-induced intestinal inflammation after the deletion of the closely related factor CDX2 the ablation of CDX1 has no effect of colitis susceptibility [45, 46]. On the other hand Rahimi and colleagues were able to show that the IL10-dependent induction of *Cd14* expression was mediated by STAT1 in human moncytic cells [47] indicating an innovative activating function of CDX1 and STAT1 in the transcriptional regulation of Cd14 in B6 mice.

In the *Cd14* allele derived from C3Bir the STAT1/CDX1 consensus sequences were disrupted creating a new SP2 binding motif introduced through a T to C substitution at position -244. We showed that the depletion of the SP2 element from the promoter by truncation and mutation led to a significant transcriptional down-regulation. Moreover, EMSA and supershift experiments proofed the protein-DNA interaction between SP2 and its C3Bir specific binding site [48]. Moreover, LeVan and colleagues reported that the ratio of Sp3 to Sp1 and Sp2 transcription factors influenced the expression of human *CD14* in monocytes and hepatocytes [49]. The retroviral transduction of primary BMMs with an empty control virus led to a reduced *Cd14* expression compared to untreated cells. Also Bösinger and colleagues found that the retroviral infection with HIV correlated with a lower expression of *Cd14* and TLR4 [46]. Compensating these inhibitory effects of the retrovirus by itself the retroviral-mediated overexpression of SP2 induced almost a doubling of *Cd14* transcription compared to wild type BMMs. Concerning the low binding affinity of SP2 to DNA [48] these results demonstrate the binding and activating capacities of SP2 to the *Cd14* promoter allele of C3Bir. While the SP1 and SP3 bind the same target gene sequences mediated by conserved carboxy-terminal zinc finger domains, SP2 binds in a complex with the transcription factor Nf-y to a trimeric histone-fold CCAAT box motif to SP2 specific target genes [50]. Genome wide binding studies of SP2 using murine embryonic fibroblasts of unclear origin did not identify *Cd14* as a major target of SP2 [39, 50,51], which might be due to the fact that most other mouse strain except C3H lack the SP2 binding site [21]. Therefrom, *Cd14* was characterized as positively regulated target gene of SP2 in C3Bir mice. SP2 may contribute to the differential expression level between B6 and C3Bir mice and was identified as innovative target for the therapy of inflammatory bowel diseases supporting the upregulation of the protective CD14 protein.

## Supporting Information

S1 Table (DOCX)

S2 Table (DOCX)

S3 Table (DOCX)
