# Supplementary material for: Transcription Factor SP2 Enhanced the Expression of Cd14 in Colitis-Susceptible C3H/HeJBir
Source: PLoS One. 2016 May 18;11(5):e0155821. doi: 10.1371/journal.pone.0155821 (PMC4871554; doi:10.1371/journal.pone.0155821)
Supplement: S1 Table — (DOCX) [file pone.0155821.s001.docx]

| **S1 Table: Full length and promotor truncation primers.**  Oligonucleotides in 5’-3’ orientation used for the amplification and cloning of full-length and truncation step fragments of *Cd14* promoters | |
| --- | --- |
| -1076for | ATT GAT GAC GAT GAC GAC GAC |
| -989forB6 | GTG ATT TGG CCA ATG TAC CAC |
| -836forB6 | TCC TAG TTG TCC TGG AAC TC |
| -722for | TTG AAA AAG CTG GGC ATG GTG G |
| -474for | AAG AGC TGG ATT TGA ACG GTG G |
| -398for | GAT CTA AGG CAC TAG GTG TG |
| -279for | CCC TAA TGC CAC TCT GAA TTC |
| -181for | GAA TTC ACA GAG GAA GGG ACA G |
| -166for | GAA GCC TTT CTC GGA GCC TA |
| -85for | TAC TTT CTC CTC AGG AGC GTG |
| +199/+203rev | GTT GTT CCT GCA ACT TCT CAG ATA GAT CTG G |
