# Supplementary material for: Transcription Factor SP2 Enhanced the Expression of Cd14 in Colitis-Susceptible C3H/HeJBir
Source: PLoS One. 2016 May 18;11(5):e0155821. doi: 10.1371/journal.pone.0155821 (PMC4871554; doi:10.1371/journal.pone.0155821)
Supplement: S3 Table — (DOCX) [file pone.0155821.s003.docx]

| **S3 Table: Site-directed mutagenesis primer for C3Bir**  Oligonucleotides in 5’-3’ orientation used for site-directed mutagenesis of the full-length *Cd14* promoter from C3Bir; mutations introduced are in bold | |
| --- | --- |
| OCT1rev | TCT TTA TTA ATT CAT TCT TCT C |
| OCT1for | ACT GAA TAA G**CG** GGG AAG AAG G |
| PPARγrev | AGG AGG ATG ACC TAT GCG CCC |
| PPARγfro | GGC ACA GA**T TAA TTA A**AT GCC ACT CTG |
| SP2rev | ATT AGG GCA TTC TGT GCC AGG AG |
| SP2for | GCC ACT CTG ATT TCT TCC TGT TTT TC**T** **TAA TTA A**C |
| SP1rev | GCA CCA GAA GCA ACA GCA ACA GCC AAG |
| SP1for | ACG CCT CTC C**G**G **AA**C CAC CAG AGC CCT |
